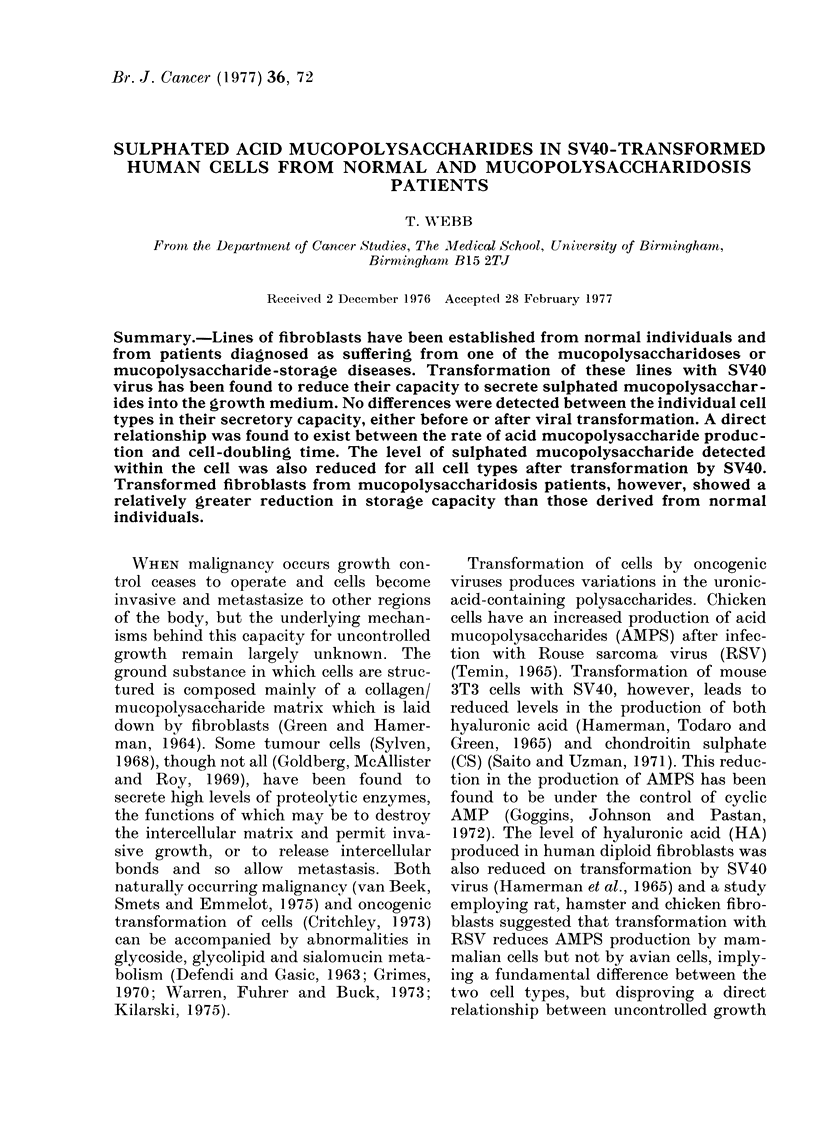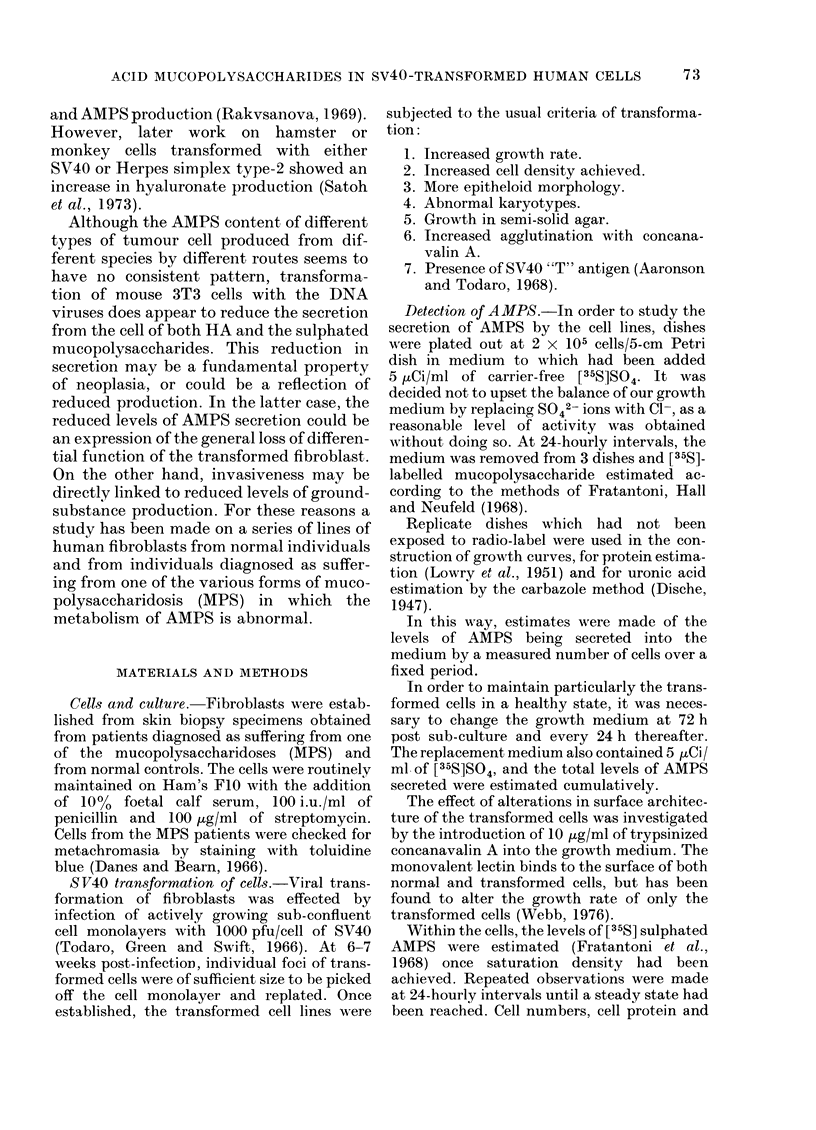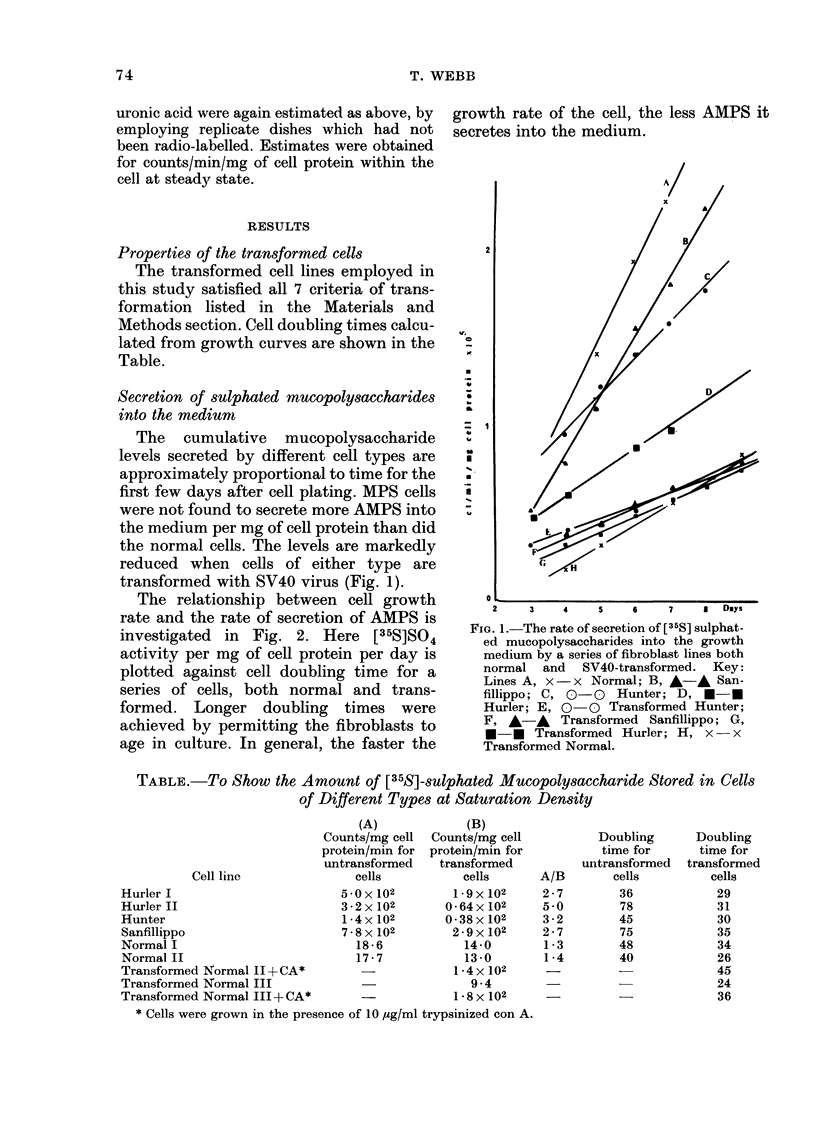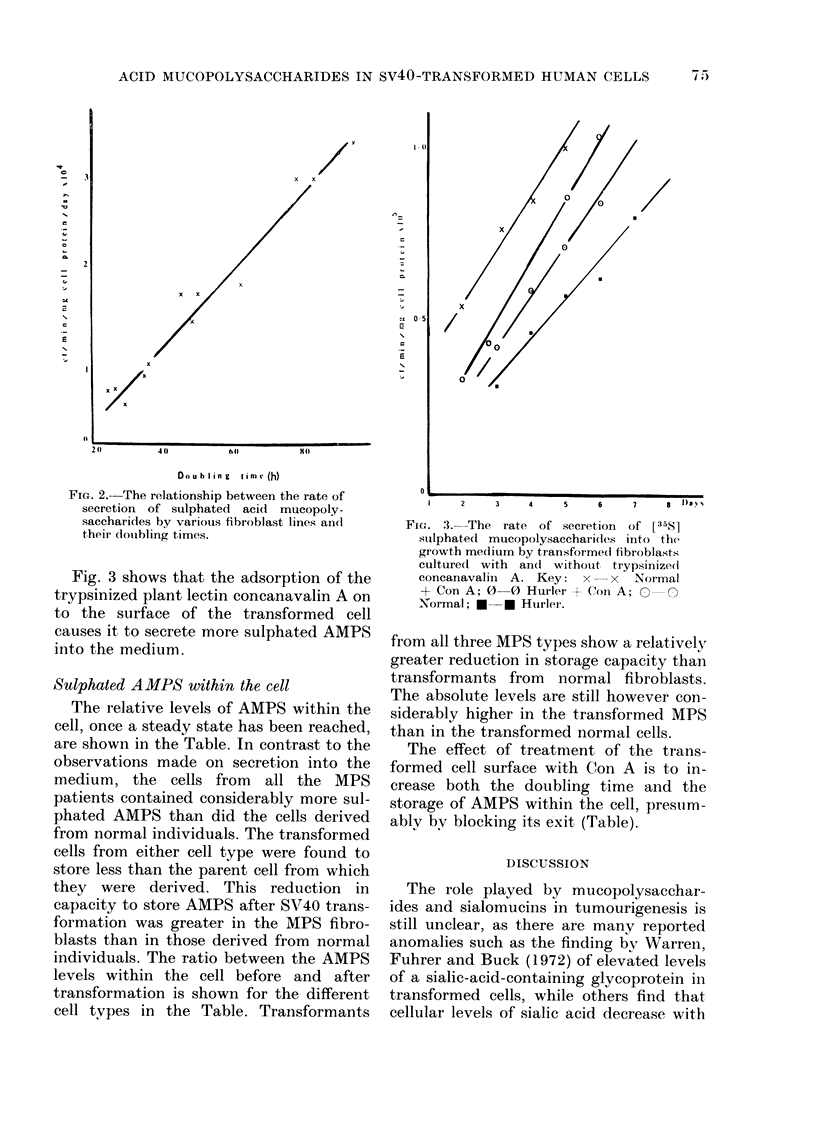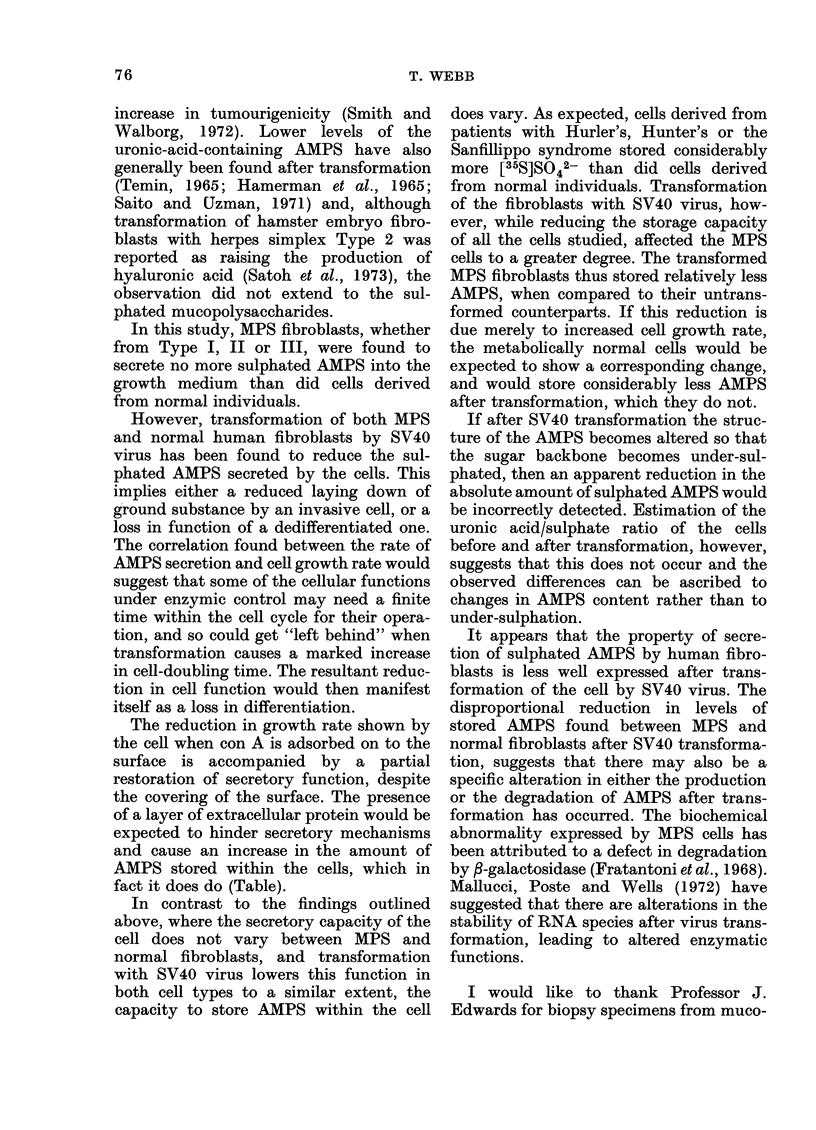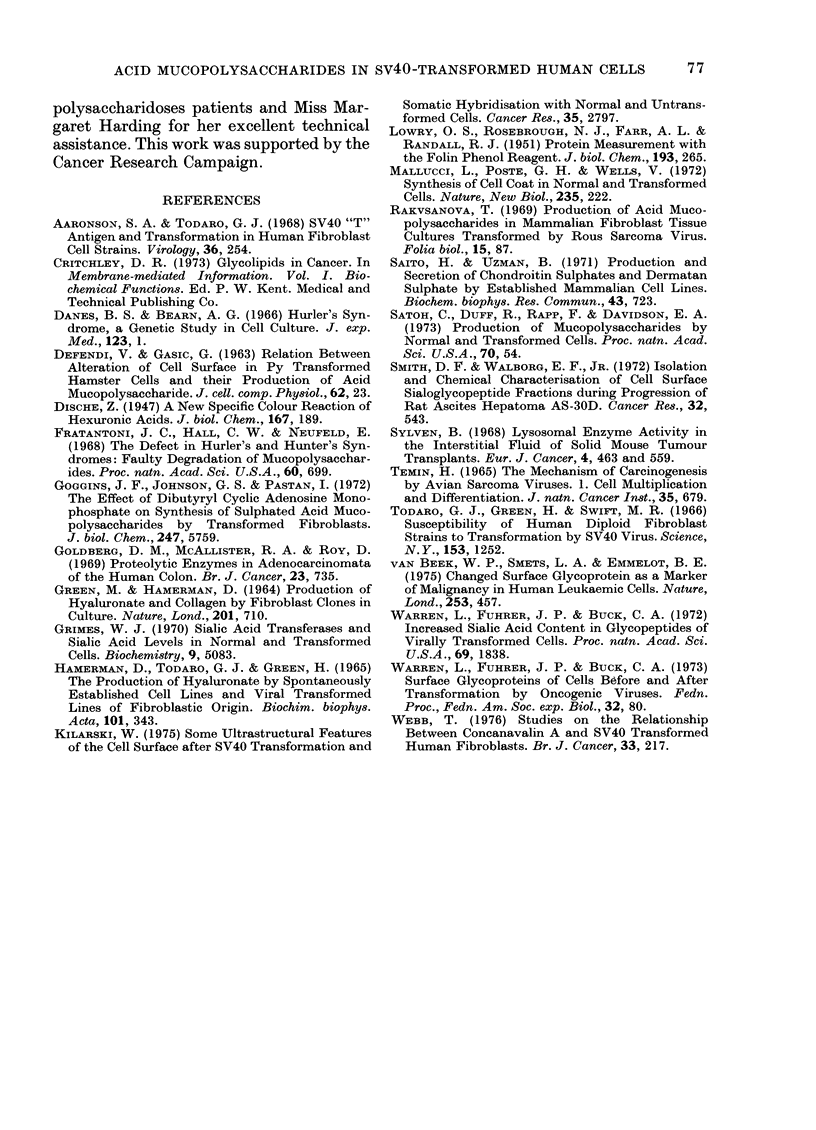# Sulphated acid mucopolysaccharides in SV40-transformed human cells from normal and mucopolysaccharidosis patients.

**DOI:** 10.1038/bjc.1977.156

**Published:** 1977-07

**Authors:** T. Webb

## Abstract

Lines of fibroblasts have been established from normal individuals and from patients diagnosed as suffering from one of the mucopolysaccharidoses or mucopolysaccharide-storage diseases. Transformation of these lines with SV40 virus has been found to reduce their capacity to secrete sulphated mucopolysaccharides into the growth medium. No differences were detected between the individual cell types in their secretory capacity, either before or after viral transformation. A direct relationship was found to exist between the rate of acid mucopolysaccharide production and cell-doubling time. The level of sulphated mucopolysaccharide detected within the cell was also reduced for all cell types after transformation by SV40. Transformed fibroblasts from mucopolysaccharidosis patients, however, showed a relatively greater reduction in storage capacity than those derived from normal individuals.


*Br. J. Cancer* (1977) **36**, 72

### SULPHATED ACID MUCOPOLYSACCHARIDES IN SV40-TRANSFORMED HUMAN CELLS FROM NORMAL AND MUCOPOLYSACCHARIDOSIS PATIENTS

T. WEBB

*From the Department of Cancer Studies, The Medical School, University of Birmingham, Birmingham B15 2TJ*

Received 2 December 1976 Accepted 28 February 1977

**Summary.—Lines of fibroblasts have been established from normal individuals and from patients diagnosed as suffering from one of the mucopolysaccharidoses or mucopolysaccharide-storage diseases. Transformation of these lines with SV40 virus has been found to reduce their capacity to secrete sulphated mucopolysaccharides into the growth medium. No differences were detected between the individual cell types in their secretory capacity, either before or after viral transformation. A direct relationship was found to exist between the rate of acid mucopolysaccharide production and cell-doubling time. The level of sulphated mucopolysaccharide detected within the cell was also reduced for all cell types after transformation by SV40. Transformed fibroblasts from mucopolysaccharidosis patients, however, showed a relatively greater reduction in storage capacity than those derived from normal individuals.**

WHEN malignancy occurs growth control ceases to operate and cells become invasive and metastasize to other regions of the body, but the underlying mechanisms behind this capacity for uncontrolled growth remain largely unknown. The ground substance in which cells are structured is composed mainly of a collagen/mucopolysaccharide matrix which is laid down by fibroblasts (Green and Hamerman, 1964). Some tumour cells (Sylven, 1968), though not all (Goldberg, McAllister and Roy, 1969), have been found to secrete high levels of proteolytic enzymes, the functions of which may be to destroy the intercellular matrix and permit invasive growth, or to release intercellular bonds and so allow metastasis. Both naturally occurring malignancy (van Beek, Smets and Emmelot, 1975) and oncogenic transformation of cells (Critchley, 1973) can be accompanied by abnormalities in glycoside, glycolipid and sialomucin metabolism (Defendi and Gasic, 1963; Grimes, 1970; Warren, Fuhrer and Buck, 1973; Kilarski, 1975).

Transformation of cells by oncogenic viruses produces variations in the uronic-acid-containing polysaccharides. Chicken cells have an increased production of acid mucopolysaccharides (AMPS) after infection with Rouse sarcoma virus (RSV) (Temin, 1965). Transformation of mouse 3T3 cells with SV40, however, leads to reduced levels in the production of both hyaluronic acid (Hamerman, Todaro and Green, 1965) and chondroitin sulphate (CS) (Saito and Uzman, 1971). This reduction in the production of AMPS has been found to be under the control of cyclic AMP (Goggins, Johnson and Pastan, 1972). The level of hyaluronic acid (HA) produced in human diploid fibroblasts was also reduced on transformation by SV40 virus (Hamerman *et al.*, 1965) and a study employing rat, hamster and chicken fibroblasts suggested that transformation with RSV reduces AMPS production by mammalian cells but not by avian cells, implying a fundamental difference between the two cell types, but disproving a direct relationship between uncontrolled growth and AMPS production (Rakvsanova, 1969). However, later work on hamster or monkey cells transformed with either SV40 or Herpes simplex type-2 showed an increase in hyaluronate production (Satoh *et al.*, 1973).

Although the AMPS content of different types of tumour cell produced from different species by different routes seems to have no consistent pattern, transformation of mouse 3T3 cells with the DNA viruses does appear to reduce the secretion from the cell of both HA and the sulphated mucopolysaccharides. This reduction in secretion may be a fundamental property of neoplasia, or could be a reflection of reduced production. In the latter case, the reduced levels of AMPS secretion could be an expression of the general loss of differential function of the transformed fibroblast. On the other hand, invasiveness may be directly linked to reduced levels of ground-substance production. For these reasons a study has been made on a series of lines of human fibroblasts from normal individuals and from individuals diagnosed as suffering from one of the various forms of mucopolysaccharidosis (MPS) in which the metabolism of AMPS is abnormal.

### MATERIALS AND METHODS

*Cells and culture.*—Fibroblasts were established from skin biopsy specimens obtained from patients diagnosed as suffering from one of the mucopolysaccharidoses (MPS) and from normal controls. The cells were routinely maintained on Ham's F10 with the addition of 10% foetal calf serum, 100 i.u./ml of penicillin and 100 $\mu$g/ml of streptomycin. Cells from the MPS patients were checked for metachromasia by staining with toluidine blue (Danes and Bearn, 1966).

*SV40 transformation of cells.*—Viral transformation of fibroblasts was effected by infection of actively growing sub-confluent cell monolayers with 1000 pfu/cell of SV40 (Todaro, Green and Swift, 1966). At 6–7 weeks post-infection, individual foci of transformed cells were of sufficient size to be picked off the cell monolayer and replated. Once established, the transformed cell lines were subjected to the usual criteria of transformation:

1. Increased growth rate.
2. Increased cell density achieved.
3. More epitheloid morphology.
4. Abnormal karyotypes.
5. Growth in semi-solid agar.
6. Increased agglutination with concanavalin A.
7. Presence of SV40 "T" antigen (Aaronson and Todaro, 1968).

*Detection of AMPS.*—In order to study the secretion of AMPS by the cell lines, dishes were plated out at $2 \times 10^5$ cells/5-cm Petri dish in medium to which had been added 5 $\mu$Ci/ml of carrier-free [$^{35}$S]$SO_4$. It was decided not to upset the balance of our growth medium by replacing $SO_4{}^{2-}$ ions with $Cl^-$, as a reasonable level of activity was obtained without doing so. At 24-hourly intervals, the medium was removed from 3 dishes and [$^{35}$S]-labelled mucopolysaccharide estimated according to the methods of Fratantoni, Hall and Neufeld (1968).

Replicate dishes which had not been exposed to radio-label were used in the construction of growth curves, for protein estimation (Lowry *et al.*, 1951) and for uronic acid estimation by the carbazole method (Dische, 1947).

In this way, estimates were made of the levels of AMPS being secreted into the medium by a measured number of cells over a fixed period.

In order to maintain particularly the transformed cells in a healthy state, it was necessary to change the growth medium at 72 h post sub-culture and every 24 h thereafter. The replacement medium also contained 5 $\mu$Ci/ml of [$^{35}$S]$SO_4$, and the total levels of AMPS secreted were estimated cumulatively.

The effect of alterations in surface architecture of the transformed cells was investigated by the introduction of 10 $\mu$g/ml of trypsinized concanavalin A into the growth medium. The monovalent lectin binds to the surface of both normal and transformed cells, but has been found to alter the growth rate of only the transformed cells (Webb, 1976).

Within the cells, the levels of [$^{35}$S] sulphated AMPS were estimated (Fratantoni *et al.*, 1968) once saturation density had been achieved. Repeated observations were made at 24-hourly intervals until a steady state had been reached. Cell numbers, cell protein and uronic acid were again estimated as above, by employing replicate dishes which had not been radio-labelled. Estimates were obtained for counts/min/mg of cell protein within the cell at steady state.

### RESULTS

### *Properties of the transformed cells*

The transformed cell lines employed in this study satisfied all 7 criteria of transformation listed in the Materials and Methods section. Cell doubling times calculated from growth curves are shown in the Table.

### *Secretion of sulphated mucopolysaccharides into the medium*

The cumulative mucopolysaccharide levels secreted by different cell types are approximately proportional to time for the first few days after cell plating. MPS cells were not found to secrete more AMPS into the medium per mg of cell protein than did the normal cells. The levels are markedly reduced when cells of either type are transformed with SV40 virus (Fig. 1).

The relationship between cell growth rate and the rate of secretion of AMPS is investigated in Fig. 2. Here [$^{35}$S]$SO_4$ activity per mg of cell protein per day is plotted against cell doubling time for a series of cells, both normal and transformed. Longer doubling times were achieved by permitting the fibroblasts to age in culture. In general, the faster the growth rate of the cell, the less AMPS it secretes into the medium.

FIG. 1.—The rate of secretion of [$^{35}$S] sulphated mucopolysaccharides into the growth medium by a series of fibroblast lines both normal and SV40-transformed. Key: Lines A, ×—× Normal; B, ▲—▲ Sanfillippo; C, ⊙—⊙ Hunter; D, ■—■ Hurler; E, ⊙—⊙ Transformed Hunter; F, ▲—▲ Transformed Sanfillippo; G, ■—■ Transformed Hurler; H, ×—× Transformed Normal.

TABLE.—*To Show the Amount of* [$^{35}$*S*]-*sulphated Mucopolysaccharide Stored in Cells of Different Types at Saturation Density*

| Cell line | (A) Counts/mg cell protein/min for untransformed cells | (B) Counts/mg cell protein/min for transformed cells | A/B | Doubling time for untransformed cells | Doubling time for transformed cells |
|---|---|---|---|---|---|
| Hurler I | $5 \cdot 0 \times 10^2$ | $1 \cdot 9 \times 10^2$ | 2·7 | 36 | 29 |
| Hurler II | $3 \cdot 2 \times 10^2$ | $0 \cdot 64 \times 10^2$ | 5·0 | 78 | 31 |
| Hunter | $1 \cdot 4 \times 10^2$ | $0 \cdot 38 \times 10^2$ | 3·2 | 45 | 30 |
| Sanfillippo | $7 \cdot 8 \times 10^2$ | $2 \cdot 9 \times 10^2$ | 2·7 | 75 | 35 |
| Normal I | 18·6 | 14·0 | 1·3 | 48 | 34 |
| Normal II | 17·7 | 13·0 | 1·4 | 40 | 26 |
| Transformed Normal II+CA* | — | $1 \cdot 4 \times 10^2$ | — | — | 45 |
| Transformed Normal III | — | 9·4 | — | — | 24 |
| Transformed Normal III+CA* | — | $1 \cdot 8 \times 10^2$ | — | — | 36 |

* Cells were grown in the presence of 10 μg/ml trypsinized con A.

FIG. 2.—The relationship between the rate of secretion of sulphated acid mucopolysaccharides by various fibroblast lines and their doubling times.

FIG. 3.—The rate of secretion of [$^{35}$S] sulphated mucopolysaccharides into the growth medium by transformed fibroblasts cultured with and without trypsinized concanavalin A. Key: ×—× Normal + Con A; Ø—Ø Hurler + Con A; ○—○ Normal; ■—■ Hurler.

Fig. 3 shows that the adsorption of the trypsinized plant lectin concanavalin A on to the surface of the transformed cell causes it to secrete more sulphated AMPS into the medium.

*Sulphated AMPS within the cell*

The relative levels of AMPS within the cell, once a steady state has been reached, are shown in the Table. In contrast to the observations made on secretion into the medium, the cells from all the MPS patients contained considerably more sulphated AMPS than did the cells derived from normal individuals. The transformed cells from either cell type were found to store less than the parent cell from which they were derived. This reduction in capacity to store AMPS after SV40 transformation was greater in the MPS fibroblasts than in those derived from normal individuals. The ratio between the AMPS levels within the cell before and after transformation is shown for the different cell types in the Table. Transformants from all three MPS types show a relatively greater reduction in storage capacity than transformants from normal fibroblasts. The absolute levels are still however considerably higher in the transformed MPS than in the transformed normal cells.

The effect of treatment of the transformed cell surface with Con A is to increase both the doubling time and the storage of AMPS within the cell, presumably by blocking its exit (Table).

### DISCUSSION

The role played by mucopolysaccharides and sialomucins in tumourigenesis is still unclear, as there are many reported anomalies such as the finding by Warren, Fuhrer and Buck (1972) of elevated levels of a sialic-acid-containing glycoprotein in transformed cells, while others find that cellular levels of sialic acid decrease with increase in tumourigenicity (Smith and Walborg, 1972). Lower levels of the uronic-acid-containing AMPS have also generally been found after transformation (Temin, 1965; Hamerman *et al.*, 1965; Saito and Uzman, 1971) and, although transformation of hamster embryo fibroblasts with herpes simplex Type 2 was reported as raising the production of hyaluronic acid (Satoh *et al.*, 1973), the observation did not extend to the sulphated mucopolysaccharides.

In this study, MPS fibroblasts, whether from Type I, II or III, were found to secrete no more sulphated AMPS into the growth medium than did cells derived from normal individuals.

However, transformation of both MPS and normal human fibroblasts by SV40 virus has been found to reduce the sulphated AMPS secreted by the cells. This implies either a reduced laying down of ground substance by an invasive cell, or a loss in function of a dedifferentiated one. The correlation found between the rate of AMPS secretion and cell growth rate would suggest that some of the cellular functions under enzymic control may need a finite time within the cell cycle for their operation, and so could get "left behind" when transformation causes a marked increase in cell-doubling time. The resultant reduction in cell function would then manifest itself as a loss in differentiation.

The reduction in growth rate shown by the cell when con A is adsorbed on to the surface is accompanied by a partial restoration of secretory function, despite the covering of the surface. The presence of a layer of extracellular protein would be expected to hinder secretory mechanisms and cause an increase in the amount of AMPS stored within the cells, which in fact it does do (Table).

In contrast to the findings outlined above, where the secretory capacity of the cell does not vary between MPS and normal fibroblasts, and transformation with SV40 virus lowers this function in both cell types to a similar extent, the capacity to store AMPS within the cell does vary. As expected, cells derived from patients with Hurler's, Hunter's or the Sanfillippo syndrome stored considerably more $[^{35}S]SO_4^{2-}$ than did cells derived from normal individuals. Transformation of the fibroblasts with SV40 virus, however, while reducing the storage capacity of all the cells studied, affected the MPS cells to a greater degree. The transformed MPS fibroblasts thus stored relatively less AMPS, when compared to their untransformed counterparts. If this reduction is due merely to increased cell growth rate, the metabolically normal cells would be expected to show a corresponding change, and would store considerably less AMPS after transformation, which they do not.

If after SV40 transformation the structure of the AMPS becomes altered so that the sugar backbone becomes under-sulphated, then an apparent reduction in the absolute amount of sulphated AMPS would be incorrectly detected. Estimation of the uronic acid/sulphate ratio of the cells before and after transformation, however, suggests that this does not occur and the observed differences can be ascribed to changes in AMPS content rather than to under-sulphation.

It appears that the property of secretion of sulphated AMPS by human fibroblasts is less well expressed after transformation of the cell by SV40 virus. The disproportional reduction in levels of stored AMPS found between MPS and normal fibroblasts after SV40 transformation, suggests that there may also be a specific alteration in either the production or the degradation of AMPS after transformation has occurred. The biochemical abnormality expressed by MPS cells has been attributed to a defect in degradation by β-galactosidase (Fratantoni *et al.*, 1968). Mallucci, Poste and Wells (1972) have suggested that there are alterations in the stability of RNA species after virus transformation, leading to altered enzymatic functions.

I would like to thank Professor J. Edwards for biopsy specimens from mucopolysaccharidoses patients and Miss Margaret Harding for her excellent technical assistance. This work was supported by the Cancer Research Campaign.

### REFERENCES

AARONSON, S. A. & TODARO, G. J. (1968) SV40 "T" Antigen and Transformation in Human Fibroblast Cell Strains. *Virology*, **36**, 254.

CRITCHLEY, D. R. (1973) Glycolipids in Cancer. In *Membrane-mediated Information. Vol. I. Biochemical Functions*. Ed. P. W. Kent. Medical and Technical Publishing Co.

DANES, B. S. & BEARN, A. G. (1966) Hurler's Syndrome, a Genetic Study in Cell Culture. *J. exp. Med.*, **123**, 1.

DEFENDI, V. & GASIC, G. (1963) Relation Between Alteration of Cell Surface in Py Transformed Hamster Cells and their Production of Acid Mucopolysaccharide. *J. cell. comp. Physiol.*, **62**, 23.

DISCHE, Z. (1947) A New Specific Colour Reaction of Hexuronic Acids. *J. biol. Chem.*, **167**, 189.

FRATANTONI, J. C., HALL, C. W. & NEUFELD, E. (1968) The Defect in Hurler's and Hunter's Syndromes: Faulty Degradation of Mucopolysaccharides. *Proc. natn. Acad. Sci. U.S.A.*, **60**, 699.

GOGGINS, J. F., JOHNSON, G. S. & PASTAN, I. (1972) The Effect of Dibutyryl Cyclic Adenosine Monophosphate on Synthesis of Sulphated Acid Mucopolysaccharides by Transformed Fibroblasts. *J. biol. Chem.*, **247**, 5759.

GOLDBERG, D. M., MCALLISTER, R. A. & ROY, D. (1969) Proteolytic Enzymes in Adenocarcinomata of the Human Colon. *Br. J. Cancer*, **23**, 735.

GREEN, M. & HAMERMAN, D. (1964) Production of Hyaluronate and Collagen by Fibroblast Clones in Culture. *Nature, Lond.*, **201**, 710.

GRIMES, W. J. (1970) Sialic Acid Transferases and Sialic Acid Levels in Normal and Transformed Cells. *Biochemistry*, **9**, 5083.

HAMERMAN, D., TODARO, G. J. & GREEN, H. (1965) The Production of Hyaluronate by Spontaneously Established Cell Lines and Viral Transformed Lines of Fibroblastic Origin. *Biochim. biophys. Acta*, **101**, 343.

KILARSKI, W. (1975) Some Ultrastructural Features of the Cell Surface after SV40 Transformation and Somatic Hybridisation with Normal and Untransformed Cells. *Cancer Res.*, **35**, 2797.

LOWRY, O. S., ROSEBROUGH, N. J., FARR, A. L. & RANDALL, R. J. (1951) Protein Measurement with the Folin Phenol Reagent. *J. biol. Chem.*, **193**, 265.

MALLUCCI, L., POSTE, G. H. & WELLS, V. (1972) Synthesis of Cell Coat in Normal and Transformed Cells. *Nature, New Biol.*, **235**, 222.

RAKVSANOVA, T. (1969) Production of Acid Mucopolysaccharides in Mammalian Fibroblast Tissue Cultures Transformed by Rous Sarcoma Virus. *Folia biol.*, **15**, 87.

SAITO, H. & UZMAN, B. (1971) Production and Secretion of Chondroitin Sulphates and Dermatan Sulphate by Established Mammalian Cell Lines. *Biochem. biophys. Res. Commun.*, **43**, 723.

SATOH, C., DUFF, R., RAPP, F. & DAVIDSON, E. A. (1973) Production of Mucopolysaccharides by Normal and Transformed Cells. *Proc. natn. Acad. Sci. U.S.A.*, **70**, 54.

SMITH, D. F. & WALBORG, E. F., JR. (1972) Isolation and Chemical Characterisation of Cell Surface Sialoglycopeptide Fractions during Progression of Rat Ascites Hepatoma AS-30D. *Cancer Res.*, **32**, 543.

SYLVEN, B. (1968) Lysosomal Enzyme Activity in the Interstitial Fluid of Solid Mouse Tumour Transplants. *Eur. J. Cancer*, **4**, 463 and 559.

TEMIN, H. (1965) The Mechanism of Carcinogenesis by Avian Sarcoma Viruses. 1. Cell Multiplication and Differentiation. *J. natn. Cancer Inst.*, **35**, 679.

TODARO, G. J., GREEN, H. & SWIFT, M. R. (1966) Susceptibility of Human Diploid Fibroblast Strains to Transformation by SV40 Virus. *Science, N.Y.*, **153**, 1252.

VAN BEEK, W. P., SMETS, L. A. & EMMELOT, B. E. (1975) Changed Surface Glycoprotein as a Marker of Malignancy in Human Leukaemic Cells. *Nature, Lond.*, **253**, 457.

WARREN, L., FUHRER, J. P. & BUCK, C. A. (1972) Increased Sialic Acid Content in Glycopeptides of Virally Transformed Cells. *Proc. natn. Acad. Sci. U.S.A.*, **69**, 1838.

WARREN, L., FUHRER, J. P. & BUCK, C. A. (1973) Surface Glycoproteins of Cells Before and After Transformation by Oncogenic Viruses. *Fedn. Proc., Fedn. Am. Soc. exp. Biol.*, **32**, 80.

WEBB, T. (1976) Studies on the Relationship Between Concanavalin A and SV40 Transformed Human Fibroblasts. *Br. J. Cancer*, **33**, 217.